# A Case-Control Study of the Association between Polymorphisms in the Fibrinogen Alpha Chain Gene and Schizophrenia

**DOI:** 10.1155/2017/3104180

**Published:** 2017-01-19

**Authors:** Wenwang Rao, Na Zhou, Huiping Zhang, Rui Liu, Shangchao Zhang, Yingying Su, Guang Yang, Yue Ma, Jieping Shi, Yaqin Yu, Qiong Yu

**Affiliations:** ^1^Department of Epidemiology and Biostatistics, School of Public Health, Jilin University, Changchun, China; ^2^Department of Pharmacy, Stomatology Hospital, Jilin University, Changchun 130041, China; ^3^Department of Psychiatry, Yale University School of Medicine, New Haven, CT, USA; ^4^VA Medical Center, VA Connecticut Healthcare System, West Haven, CT, USA

## Abstract

Our previous studies using the mass spectrum analysis provided evidence that fibrinopeptide A (FPA) could be a potential biomarker for schizophrenia diagnosis. We sought further to demonstrate that variants in the fibrinogen alpha chain gene* (FGA)* coded FPA might confer vulnerability to schizophrenia. 1,145 patients with schizophrenia and 1,016 healthy volunteers from the Han population in Northeast China were recruited. The association of three tag single nucleotide polymorphisms (SNPs) (rs2070011 in the 5′UTR, rs2070016 in intron 4, and rs2070022 in the 3′UTR) in* FGA* and schizophrenia was examined using a case-control study design. Genotypic distributions of these three SNPs were not found to be significantly different between cases and controls (rs2070011: *χ*^2^ = 1.28, *P* = 0.528; rs2070016: *χ*^2^ = 4.11, *P* = 0.128; rs2070022: *χ*^2^ = 1.23, *P* = 0.541). There were also no significant differences in SNP allelic frequencies between cases and controls (all *P* > 0.05). Additionally, the frequency of haplotypes consisting of alleles of these three SNPs was not significantly different between cases and healthy control subjects (global *χ*^2^ = 9.27, *P* = 0.159). Our study did not show a significant association of* FGA* SNPs with schizophrenia. Future studies may need to test more* FGA* SNPs in a larger sample to identify those SNPs with a minor or moderate effect on schizophrenia.

## 1. Introduction

Schizophrenia (SZ, OMIM181500), which is characterized by delusions, hallucinations, altered cognition, emotional reactivity, and disorganized behavior [1], remains one of the most severe and heterogeneous neuropsychiatric disorders. It affects approximately 1% of the population worldwide. Owing to the pervasiveness of associated deficits and frequently life-long course, the burden of schizophrenia is ranked among the top ten diseases in the world [2]. Despite vigorous studies on schizophrenia over the past century, the etiology of schizophrenia remains obscure and available treatments for schizophrenia are only modestly effective [3].

Our recent study demonstrated that fibrinopeptide A (FPA) was a potential biomarker for diagnosis of schizophrenia using the mass spectrum analysis [4]. First, our study built the diagnosis model by analyzing serum protein expression differences between 166 schizophrenic patients and 201 healthy controls. Second, we validated this model in newly recruited 76 schizophrenic patients and 103 healthy volunteers. Third, we assessed the ability of the pattern in differentiating schizophrenia from other chronic diseases by testing the model in another set of a sample consisting of 44 cases and 41 hypertensive or diabetic patients. Fourth, our study observed a number of peaks that were different between patients with first-episode schizophrenia and healthy volunteers via the diagnosis model. When* m/z* 1206.58 of the peptide was served as an independent biomarker to differentiate patients with schizophrenia from healthy volunteers, the area under the curve (AUC) was 0.981 in receiver operating characteristic (ROC) curve analysis. While* m/z* 1206.58 of the peptide was utilized to differentiate schizophrenic patients from patients with other chronic diseases, the AUC reached 0.999 in ROC curve analysis. Finally, through the MALDI-TOF/TOF-MS analysis [5], the peptide with* m/z* 1206.58 was characterized as FPA.

Given that the level of FPA was significantly different between schizophrenic patients and healthy individuals [4], we speculated whether some polymorphisms located in the* FGA* gene may have regulated the expression of* FGA* gene. Hence, our research group selected three* FGA* tagSNPs and analyzed their association with schizophrenia in the Chinese Han population.

## 2. Materials and Methods 

### 2.1. Study Population

A total of 1,145 schizophrenia patients (mean age = 36 ± 11 years; 49.2% males) were recruited from the Changchun Mental Hospital of Jilin University in Northeast of China. Two independent experienced psychiatrists interviewed each patient and made the diagnosis based on the International Statistical Classification of Disease and Related Health Problems, Tenth Revision. The concordant diagnosis was obtained between two raters, with an interrater reliability for diagnostic consensus *k* > 0.9. Healthy control subjects (mean age = 34 ± 12 years; 53.4% males), consisting of 1,016 healthy volunteers without a past and present history of psychiatric disorders, were recruited from the Health Medical Center, located at the First Hospital Affiliated to Jilin University in Northeast China.

All subjects gave written informed consent, which was approved by the ethics committee of the School of Public Health, Jilin University. Each participant was Han Chinese living in Jilin Province of China. They attended this study on a voluntary basis and voluntarily offered blood samples for the genotyping analysis.

### 2.2. DNA Extraction and SNP Genotyping

Genomic DNA was extracted from peripheral blood lymphocytes using the ClotBlood DNA Kit (Cwbio, Beijing) and detected using an ultraviolet spectrophotometer (Beckman, USA), OD260/OD280: 1.6–1.9.

We selected three tagSNPs located in the fibrinogen alpha chain gene* (FGA)* by Haploview 4.2 using genotype data from Genome Browser release #27 in the HapMap Project (http://www.hapmap.org).* FGA* is mapped to Chromosome 4q28 and contains six exons. These three tagSNPs included rs2070011 (in the 5′UTR of* FGA*), rs2070016 (in intron 4 of* FGA*), and rs2070022 (in the 3′UTR of* FGA*). The primers for genotyping these three tagSNPs were designed using the AssayDesigner3.1 and are listed in Table 1.

SNP genotyping was determined by the allele-specific matrix-assisted laser desorption/ionization-time-of-flight mass spectrometry (MALDI-TOF-MS) using the Sequenom MassARRAY platform in Bio-X Institutes, Beijing [6]. Using a MassARRAY Nanodispenser (Sequenom, San Diego, CA, USA), standardized genotyping reactions were dispensed onto a 384-well spectroCHIP [7]. The repeated control samples were set in every genotyping plate and the concordance was more than 99%.

### 2.3. Statistical Analysis

Sex differences were compared between cases and controls by Pearson's *χ*^2^ tests. The Hardy-Weinberg equilibrium (HWE) was examined by the Chi-square (*χ*^2^) goodness-of-fit test. The* FGA* SNP allele frequencies and genotype distributions were analyzed using the Chi-square (*χ*^2^) test. Pairwise linkage disequilibrium (LD) between* FGA* SNPs was operated using Haploview 4.2 [8]. The haplotype analysis was executed using UNPHASED 2.404 [9], and 10,000 permutations were performed to adjust for multiple testing. The power defined that the probability that the test correctly rejects the null hypothesis (H0) when the alternative hypothesis (H1) is true of the sample was calculated using Quanto 1.2.4 Software [10]. We hypothesized the known risk allele frequencies and a schizophrenia population prevalence of 0.01 as well as analyzing log-additive, recessive, and dominant models, respectively.

All statistical analysis was carried out using SPSS version 21.0 (IBM SPSS, IBM Corp, Armonk, NY, USA). *P*  values were two-tailed, and *P* < 0.05 was considered to be the suggestive association level.

## 3. Results

### 3.1. Demographic Characteristics and HWE Test Results

No significant sex differences were found between cases with schizophrenia and healthy controls (*χ*^2^ = 3.76, *P* = 0.053). No deviation from HWE was detected in both cases and control groups for the three tagSNPs (all *P* > 0.05) except for rs2070011 and rs2070016 in the control group (*χ*^2^ = 5.59, *P* = 0.018; *χ*^2^ = 5.71, *P* = 0.017). We randomly chose 10% of the samples from the control group to replicate the genotyping results. The genotyping accuracy rate was more than 99%.

### 3.2. The Allele and Genotype Analysis

Table 2 shows genotype distributions and allele frequencies of three* FGA* tagSNPs in schizophrenic patients and healthy controls. Between schizophrenic patients and controls, no significant difference was observed in genotype distributions of the three tagSNPs (all *P* > 0.05). With regard to allele frequencies of the three tagSNPs, we found no significant differences between schizophrenic patients and healthy controls (all *P* > 0.05). In addition, inheritance modeling did not show significant differences in genotype frequency distributions between two cases and control for all three tagSNPs (all *P* > 0.05).

### 3.3. The Haplotype Analysis

**Figure 1:**
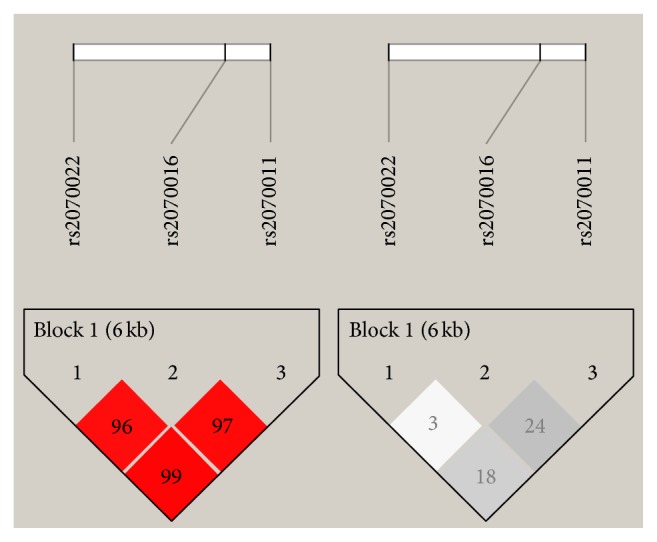
Linkage disequilibrium (LD) of three* FGA* tagSNPs. The linkage disequilibrium (LD) between pairwise SNPs, using *D*′ (left, red color) and *r*^2^ (right, gray color) values, is shown for all subjects. High levels of LD are represented by increasing scale intensity from 0 to 100, as shown by the bars.

The LD patterns of the three* FGA* SNPs are shown in Figure 1. The three tagSNPs were in LD. In view of the strong linkage disequilibrium in the present subjects, only eight 3-SNP common haplotypes (rs2070011, rs2070016, and rs2070022) were estimated to have a frequency of >1%. There were no significant differences observed in overall haplotype frequencies between schizophrenic patients and healthy controls (global *χ*^2^ = 9.27, df = 6, and *P* = 0.159) through 10,000 permutation corrections. In addition, we did not notice significant differences in individual haplotypes between cases and controls (ACC: *χ*^2^ = 1.98, *P* = 0.160; ATC: *χ*^2^ = 0.22, *P* = 0.638; ATT: *χ*^2^ = 1.50, *P* = 0.221; GCC: *χ*^2^ = 0.15, *P* = 0.696; GCT: *χ*^2^ = 2.37, *P* = 0.124; GTC: *χ*^2^ = 1.60, *P* = 0.205; and GTT: *χ*^2^ = 1.79, *P* = 0.181).

### 3.4. The Power Analysis

Our sample had a power of 0.90–0.99 for rs2070011, 0.30–0.99 for rs2070016, and 0.22–0.99 for rs2070022, respectively, to reveal an association of* FGA* tagSNPs and schizophrenia. We examined dominant, recessive, and log-additive polymorphic inheritance in schizophrenia with an odds ratio (OR) ranging from 1.4 to 2.0 (*α* = 0.05, two-tailed test).

## 4. Discussion

A number of studies have revealed the association between variants of a list of genes and schizophrenia [11]. To date, the association of* FGA* variants with schizophrenia little has been reported.* FGA* encodes the alpha component of fibrinogen that is a blood-borne glycoprotein comprising three pairs of nonidentical polypeptide chains. To our knowledge, this is the first study that investigated the association of* FGA* variants and schizophrenia in the Chinese Han population in Northeast China. Our findings suggested that* FGA* polymorphisms might not be associated with the susceptibility to schizophrenia.

At present, our study did not observe a significant association of* FGA* polymorphisms and schizophrenia. This is consistent with the results from previous studies. For example, in the study by Liu et al. [12], no significant difference was obtained when the number of core unit repeats of an* FGA* short tandem repeat (STR) polymorphism was compared between schizophrenia patients and healthy controls. Jungerius et al. [13] also reported negative associations of* FGA* variants and schizophrenia (*P* = 0.0143, by rs2070022; *P* = 0.0972, by rs2070016). In view of the relatively large sample size and our high statistical power, these results from our study were reliable and accurate and repeatable. However, the negative findings for the three possibly functional* FGA* SNPs are beyond our expectation that the FPA has the potential clinical diagnostic value as a biomarker of schizophrenia. One of the explanations causing this discrepancy is that epigenetic regulation may play important roles in the formation and development of* FPA* [14]. More specifically, various environmental factors, particularly when occurring during development, have been claimed to produce long-lasting epigenetic changes at the* FGA* gene, thereby affecting availability and function of FPA [15]. However, this is only our speculation and further detailed investigations need to be explored.

Our study has three major limitations. First, our study had eliminated genotyping errors and chance or failure of the assumptions of HWE owing to departure from HWE. Thus, the explanation of the association between* FGA *variants and schizophrenia was rational [16–18]. Second, our present study only genotyped three* FGA* tagSNPs, nevertheless, in view of the fact that the total* FGA* variants contain more than 500 polymorphic loci. Hence, it is necessary to fine-map* FGA* to identify schizophrenia-associated* FGA* SNPs. Third, although our previous study detected FPA serum levels from patients with schizophrenia, we did not note enough data information. Thus, we cannot carry out further analyses to evaluate the role of the selected SNPs in FPA serum levels.

## 5. Conclusions 

To sum up, the present study did not provide evidences to support the fact that* FGA *variants might influence the susceptibility to schizophrenia in the Han Chinese population from Northeast China. However, the current association findings remain preliminary until replicated in other independent larger samples in different ethnicities.

## Figures and Tables

**Table 1:** Primers for polymerase chain reaction.

SNPs	Primer sequence (5′-3′)
rs2070011	F: 5′-ACGTTGGATGGATTAACCAAACCTCTTGCAG-3′,
	R: 5′-ACGTTGGATGGCACTCCAGCTGAAAGAAAG-3′
rs2070016	F: 5′-ACGTTGGATGGAGAAACTCTGGAATGAGGG-3′,
	R: 5′-ACGTTGGATGAACCAGGCTCCTGAGTATTG-3′
rs2070022	F: 5′-ACGTTGGATGCTCTCTGCAACCTTGAAGAC-3′,
	R: 5′-ACGTTGGATGTGCTTTCCAATGGCATTATG-3′

**Table 2:** Comparison of allele frequencies and genotype distributions of three *FGA* tagSNPs between schizophrenic patients and healthy controls.

tagSNP^a^	Group (*n*)^b^	Genotypic association (%)					Allelic association (%)			
		1/1	1/2	2/2	*χ* ^2^	*P*	1	2	*χ* ^2^	*P*
rs2070011	SCZ (1144)	332	586	226	1.28	0.528	1250	1038	0.36	0.550
		(29.0)	(51.2)	(19.8)			(54.6)	(45.4)		
	NC (1007)	272	538	197			1082	932		
		(27.0)	(53.4)	(19.6)			(53.7)	(46.3)		

rs2070016	SCZ (1139)	33	318	788	4.10	0.128	384	1894	0.35	0.554
		(2.9)	(27.9)	(69.2)			(16.9)	(83.1)		
	NC (966)	19	301	646			339	1593		
		(2.0)	(31.1)	(66.9)			(17.5)	(82.5)		

rs2070022	SCZ (1144)	845	277	22	1.23	0.541	1967	321	1.22	0.269
		(73.9)	(24.2)	(1.9)			(86.0)	(14.0)		
	NC (1002)	760	226	16			1746	258		
		(75.8)	(22.6)	(1.6)			(87.1)	(12.9)		

^a^The alleles of rs2070011 are A and G, the alleles of rs2070016 are C and T, and the alleles of rs2070022 are C and T.

^b^SCZ: schizophrenia; NC: normal control.
